# Utilization of Zeolitic Waste in Alkali-Activated Biomass Bottom Ash Blends

**DOI:** 10.3390/molecules25133053

**Published:** 2020-07-03

**Authors:** Danutė Vaičiukynienė, Dalia Nizevičienė, Agnė Mikelionienė, Algirdas Radzevičius

**Affiliations:** 1 Faculty of Water and Land Management, Vytautas Magnus University Agriculture Academy, Studentu st. 11, LT-53361 Akademija, Lithuania; agne.mikelioniene@gmail.com (A.M.); algirdas.radzevicius@vdu.lt (A.R.); 2 Faculty of Electrical and Electronics Engineering, Kaunas University of Technology, Studentu st. 48, LT-51367 Kaunas, Lithuania; dalia.nizeviciene@ktu.lt

**Keywords:** alkali-activated biomass ash, zeolitic waste with NH_4_Cl, alkali-activated binder

## Abstract

This study aims to investigate the effects of ammonium-bearing zeolitic waste (FCC) on alkali-activated biomass bottom ash (BBA). FCC was obtained from the oil-cracking process in petroleum plants. In this study, two types of production waste were used: biomass bottom ash and ammonium-bearing zeolitic waste. These binary alkali-activated FCC/BBA blends were investigated using X-ray diffraction (XRD), Fourier transform infrared (FTIR) and scanning electron microscopy (SEM) methods. The compressive strength of the hardened samples was evaluated. The results show that the samples made from alkali-activated BBA biomass bottom ash had low (8.5 MPa) compressive strength, which could be explained with low reactive BBA and insufficient quantities of silicon and aluminum compounds. The reactivity of BBA was improved with incorporating zeolitic waste as an aluminosilicate material. This zeolitic waste was first used for ammonium sorption; then, it was incorporated in alkali-activated samples. Additional amounts of hydrated products formed, such as calcium silicate hydrate, calcium aluminum silicate hydrate and calcium sodium aluminum silicate hydrate. The silicon and aluminum compound, which varied in zeolitic waste, changed the mineral composition and microstructure of alkali-activated binder systems. NH_4_Cl, which was incorporated in the zeolitic waste, did not negatively affect the compressive strength of the alkali-activated BBA samples. This investigation proved that waste materials can be reused by producing alkali-activated binders.

## 1. Introduction

Statistics studies show that the consumption of biofuels increased in the past ten years in Lithuania. Large amounts of ash were generated during biomass combustion processes, which negatively affected the environment. To date, only a small amount of biomass ash is reused, and the largest part of this type of ash is disposed in landfills. One of the methods to reuse biomass ash is to produce binders (based on alkali activation). Biomass ash is used as the aluminosilicate initial material. Rajamma et al. [1] investigated biomass fly ash–metakaolin blends activated with sodium hydroxide and sodium silicate solutions. The compressive strength of the mortar samples reached 38 MPa, and metakaolin had a positive effect in these alkali-activated blends. In another study [2], an alkali-activated binder was produced using the aluminosilicate ternary system: blast furnace slag, biomass ash and rice husk ash. The strength of the mortar samples was 40–70 MPa. Zhu et al. [3] used incineration bottom ash as the aluminosilicate precursor to prepare the alkali-activated binder. Thus, this binder had 17 wt% of aluminosilicate gel and approximately 20 wt% calcium-containing phases C-S-H as the reaction products.

The next aluminosilicate raw material in this work is zeolitic waste from the oil-cracking process in petroleum plants. This zeolitic waste (spent fluid catalytic cracking (FCC) catalyst) is the fine powder of zeolite Y. Few studies used zeolitic waste as the alkali-activated binder. Ruiz et al. [4] produced an alkali-activated binder using this zeolitic waste. A compressive strength of 24.7 MPa was reached after seven days of hardening. Rodríguez et al. [5] suggested valorizing the FCC catalyst in the alkali-activated binder. During alkali activation, the precursor of the zeolitic phase converted to a highly Al-substituted aluminosilicate binder gel. Tashima et al. [6] published information about the geopolymer based on FCC. They stated that the geopolymeric mortar samples reached 68 MPa of compression strength after three days of curing at 65 °C. The interest of using a spent FCC catalyst in the preparation of alkali-activated binders in spite of being regenerated and used again in the process could be the increase of reactivity of the aluminosilicate precursor. In this case, the blend was formed from the spent FCC catalyst and biomass bottom ash. 

It was proven that the main properties of alkali-activated binding materials are strongly influenced by the composition of the precursors. These precursors usually form from materials rich in calcium, such as granulated blast furnace slag, gypsum, phospogypsum, Portland cement, limestone or high calcium fly ash, and materials rich in aluminum and silicon, such as low calcium fly ash, metakaolin and FCC. After the alkali activation, calcium silicate hydrate (C–S–H) forms from the precursors rich in calcium, and sodium aluminosilicate hydrates (N–S–A–H) forms from aluminosilicate precursors low in calcium [7]. Ye et al. [8] investigated the interaction between the calcium-rich precursor (slag) and silicon with aluminum-rich (fly ash) precursor during alkali-activation. The hydration products consisted of various types of C–A–S–H and N–A–S–H gels and the crystalline phases. The calcium from slag can incorporate into the N–A–S–H and N–(C)–A–S–H forms, and finally, it could convert to crystalline zeolites. Another reaction is possible in the N–A–S–H system when sodium is substituted with calcium and forms the C–A–S–H gel.

In this study, the reactivity of biomass bottom ash in the reaction of alkali activation can be improved using blends based on aluminosilicate. The ammonium-bearing zeolitic waste as the aluminosilicate precursor was used. Zeolitic waste has been investigated as a suitable material for the blends with biomass bottom ash because of its high silica and alumina composition. At the beginning, zeolitic waste was used for ammonium sorption [9]; then, when this zeolitic waste stopped working as a sorbent, it was incorporated in alkali-activated binder systems. 

The chemicals in Portland cement systems can be used for the accelerating setting alkali-activated materials. Many inorganic salts affect the setting of alkali-activated cements. Myrdal et al. [10] classified various admixtures according to the setting times. One of the cement setting accelerators is ammonium chloride (NH_4_Cl). These accelerators with chloride can stimulate corrosion in concrete reinforcements. However, it remains an acceptable and highly effective admixture, especially for unreinforced concrete or composite materials with lignocellulose materials. It is possible to use a NH_4_Cl cement-setting accelerator in cement-bonded composites with wood waste. Frybort et al. [11,12] determined that the addition of 2% NH_4_Cl improved the pull-out strength strands, which were embedded in the hardened cement paste. The addition of NH_4_Cl accelerates the cement-setting and curing times and improves the mechanical properties of Portland cement composite materials. This positive effect is associated with the neutralization of aggressive extractives from wooden particles.

Several admixtures can be used in the fly ash geopolymer to control the setting time. Fawzi et al. [13] investigated the influence of ammonium hydroxide (NH_4_OH) solutions on the Portland cement concrete compressive and flexural strengths. The best mechanical properties of the concrete samples were obtained with 1% of the ammonia solution. In this case, the compressive strength increased from 33 MPa (without the ammonia solution) to 37 MPa (with the ammonia solution) after 28 days. The flexural strength had the same tendency: it increased the hydration by 1.58 times. Similar results were published by Webb et al. [14]. The addition of ammonium hydroxide significantly increased the flexural strength of hardened cement paste and mortar based on Portland cement. Chang et al. [15] stated that the NH_4_Cl accelerator was effective in various combinations of cement, fly ash, slag and shell-lime. In all samples where the NH_4_Cl accelerator was used, a higher compressive strength was achieved. 

In this study, two types of production waste were used as the precursor: biomass bottom ash and ammonium-bearing zeolitic waste (FCC). The aim was to investigate the influence of this zeolitic waste on the main properties of alkali-activated biomass bottom ash. 

## 2. Results and Discussion

**Figure 1:**
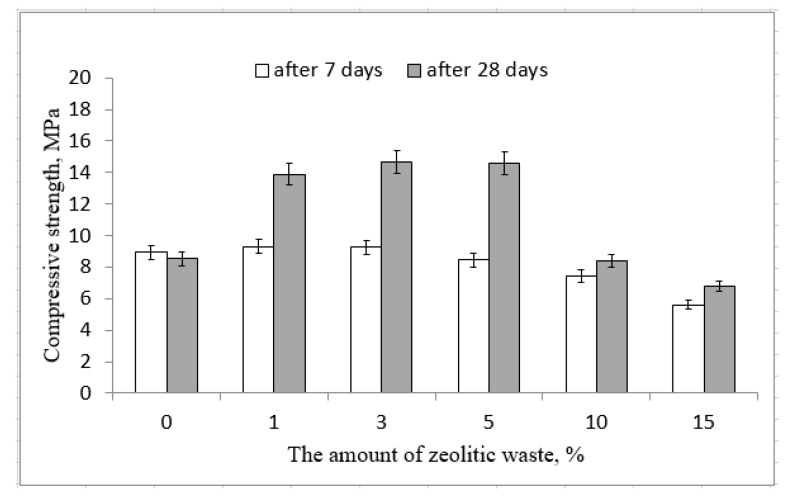
Effect of zeolitic waste on the compressive strength of alkali-activated biomass bottom ash.

The mechanical properties of binders are very important properties. Usually, alkali-activated biomass bottom ash has significantly lower mechanical properties than the fly ash-based [16,17]. This type of binder compression strength depends on the amount of zeolitic waste. After an early time of hydration (seven days), the effect of zeolitic waste is insignificant. In the system with incorporated zeolitic waste, the compression strength slightly increased from 8.9 MPa to 9.3 MPa without zeolitic waste and by 3% of this type of aluminosilicate material, respectively (Figure 1). With more zeolitic waste, the compressive strength of the samples slightly decreased. 

**Figure 2:**
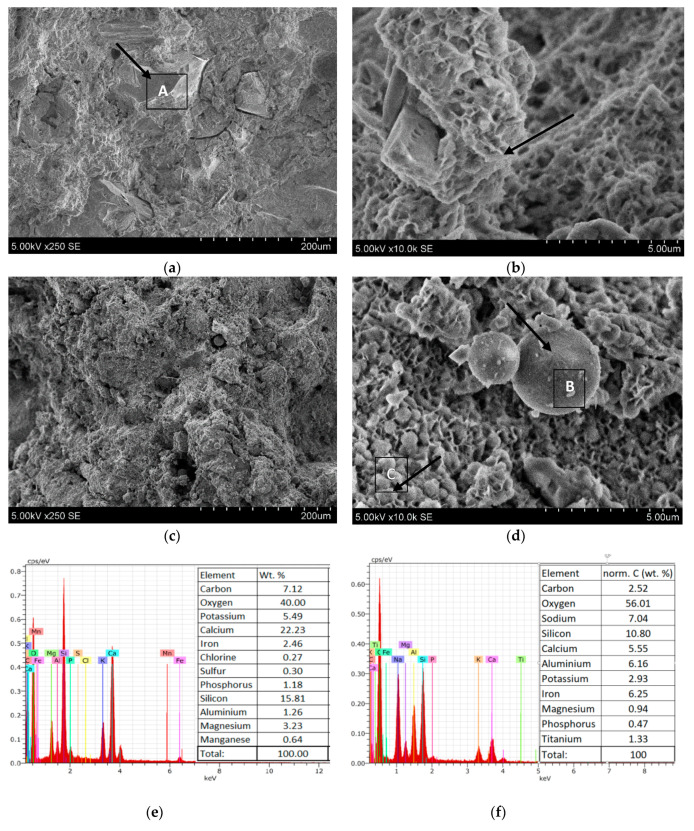

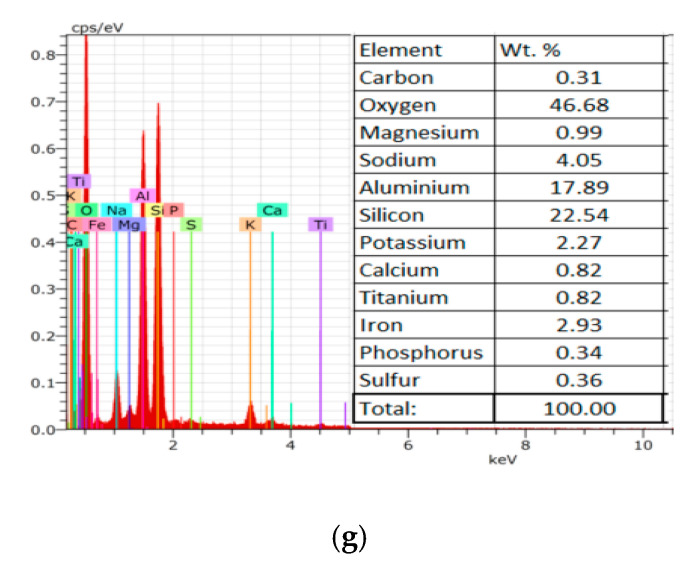
Microstructures of the alkali-activated biomass bottom ash without (**a**,**b**) and with 15% of zeolitic waste (**c**,**d**) and the EDX spectra (**e**–**g**).

The strength of alkali-activated biomass bottom ash increased when the hydration time increased from 7 to 28 days. The more significant impact of zeolitic waste on the compression strength was after 28 days of hydration. The highest compression strength of the samples was 14.7 MPa for samples with 3% of zeolitic waste, while the reference samples had 8.5 MPa. Additional amounts of silica and alumina from zeolitic waste had positive effects on the formation of alkali activation products (calcium silicate hydrate, calcium aluminum silicate hydrate and calcium sodium aluminum silicate hydrate), which are responsible for the strength development [18]. Thus, the samples with 1%, 3% and 5% of zeolitic waste had higher compressive strengths than the samples without zeolitic waste. However, the incorporation of more zeolitic waste (15%) was connected with a slightly lower compressive strength (7.0 MPa) than the reference sample without zeolitic waste. This decrease led to the overconcentration of Al and Si ions in the mixtures. In the alkali-activated systems, calcium and sodium ions were deficient, and the formation of CASH and NASH products slowed down. This statement was confirmed by the SEM analysis. It was possible to detect the unreacted zeolitic particles in the microstructure of the alkali-activated biomass bottom ash (Figure 2c,d). Similar results were obtained by Boonserm et al. [19].

The effect of the presence of ammonium chloride in the zeolitic waste dis not negatively affect the compressive strength of the samples. When the amount of zeolite waste was very high, the compressive strength was almost equal to that of the reference samples (with 10%) or it was slightly lower (with 15%) (Figure 1). The use of cement-setting accelerator ammonium chloride (NH_4_Cl) was not recommended due to the potential corrosion issues caused by chloride in reinforced concrete. It was recommended to use only for plain concrete without reinforcement. 

**Figure 3:**
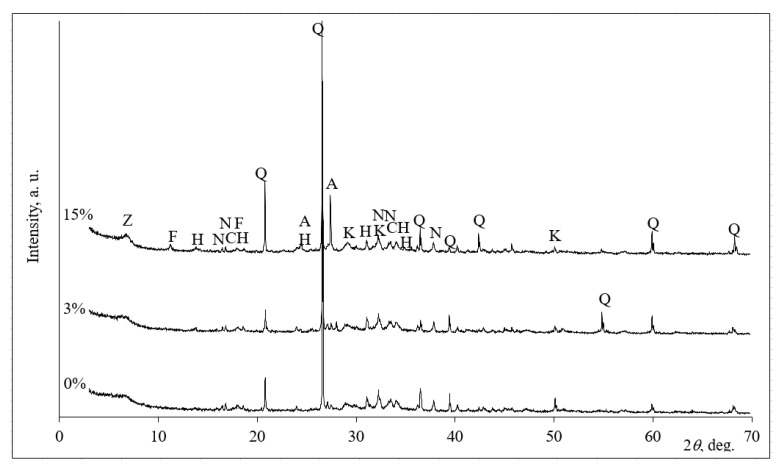
XRD analysis of alkali-activated biomass bottom ash without zeolitic waste (0%) and with (3% and 15%) zeolitic waste. Notes: Q—(83–539) quartz SiO_2_, CH–(81–2040) calcium hydroxide Ca(OH)_2_, N—(70–845) sodium carbonate hydrate Na_2_CO_3_(H_2_O), K—(33–306) calcium silicate hydrate Ca_1.5_SiO_3.5_ H_2_O, Z—(39–222) sodium aluminum silicate hydrate Na_96_Al_96_Si_96_O_384_216H_2_O, F—(78–1219) Friedel’s salt Ca_2_Al(OH)_6_Cl(H_2_O)_2_, H—(76–1639) hydrosodalite Na_8_Al_6_Si_6_O_24_(OH)_2_(H_2_O)_2_ and A—(75–1631) anorthoclase (Na,K)(Si_3_Al)O_8_.

The XRD patterns in Figure 3 show the mineral composition of the alkali-activated biomass bottom. After alkali activation, the compounds of quartz, calcium hydroxide and anorthoclase were stable, and they were originally present in the biomass bottom ash. In the samples without zeolitic waste (0%), the main changes that occurred during alkali activation were the formation of calcium silicate hydrate and sodium aluminum silicate hydrate [20].

The zeolitic waste affects the mineral composition of the samples after alkali activation. In these cases, (Figure 3, 3% and 15% diffractograms) calcium silicate hydrate, two types of sodium aluminum silicate hydrates and Friedel’s salt were formed. During the alkali activation, aluminosilicate precursors based on biomass bottom ash that was rich in calcium and zeolitic waste that was rich in aluminum and silicon compounds stimulated the formation of hydrosodalite, and the amount of sodium aluminum silicate hydrate increased. The peaks related to the sodium aluminum silicate hydrate were more intensive for the samples with zeolitic waste than those without zeolitic waste (Figure 3, 0% and 3% diffractograms). Consequently, during alkali activation, the slightly higher amount of newly formed minerals positively affected the compressive strength of the samples (Figure 1). It was detected, a slightly lower compressive strength with 15% of zeolitic waste was determined due to the formation the highest amount of CASH and NASH products (Figure 3, 15% diffractogram).

**Figure 4:**
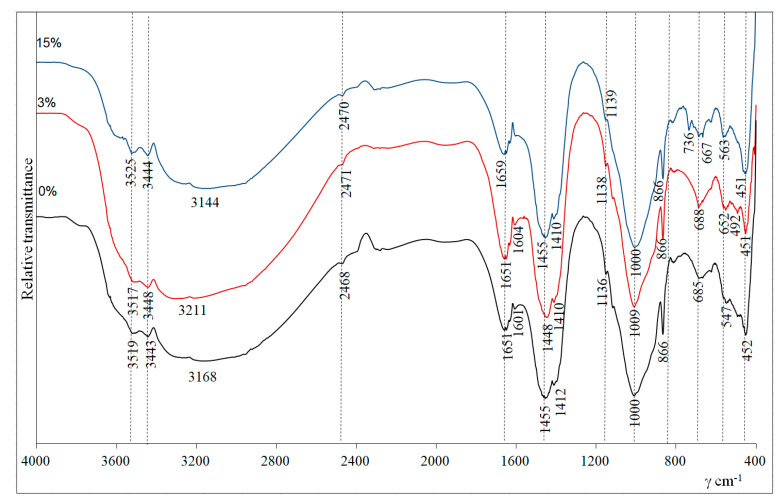
Fourier-transform infrared FTIR analysis of alkali-activated biomass bottom ash without zeolitic waste (0%) and with (3% and 15%) zeolitic waste.

XRD and FTIR spectroscopy were used to characterize the mineral composition of the alkali-activated biomass bottom ash. Figure 4 shows the Fourier-transform infrared (FTIR) analysis of the alkali-activated biomass bottom ash. According to Part et al. [21], the bands at 1651–1659 cm^−1^ and 3443–3448 cm^−1^ formed after the alkali activation of the aluminosilicate precursors and can confirm the stretching vibration of –OH and bending vibration of O–H–O. The most intense aforementioned bands (1651–1659 cm^−1^ and 3443–3448 cm^−1^) have alkali-activated biomass bottom ash with 3% zeolitic waste compared to the bands of 0% and 15% curves (Figure 4). The strong and wide bands at 3144–3211 cm^−1^ could be assigned to the stretching vibrations of hydroxyl groups (–OH) of water. The peaks in the rage of 1000–1009 cm^−1^ were assigned to the aluminosilicate gel, i.e., Si-O-Si(Al) formed bonds after alkaline activation. This peak was the highest in the samples, with 3% of zeolitic waste. Consequently, the highest amount of new aluminosilicate phases formed during alkaline activation of biomass bottom ash with 3% of zeolitic waste, and the same tendency was observed by XRD. These newly formed hydrates positively affected the compressive strength of the samples (Figure 1).

The increased amount of gel can result in the highest compressive strength [22]. The peaks at 2468–2471, 1448–1455, 866 and 736 cm^−1^ are attributed to the presence of vibrations of CO_3_^2^ (carbonates). Similar trends were observed by Zhu et al. [23]. The bands at approximately 1136–1139, 780, 562–563 and 451–452 cm^−1^ were detected and could be attributed to vibrations of Si-O (quartz) [24]. The peaks at 3517–525 cm^−1^ are due to the OH^−^ stretching mode of calcium hydroxide [25]. This result is consistent with the XRD result (Figure 3).

The microstructure of alkali-activated biomass bottom ash is shown in Figure 2. In the samples without zeolitic waste, nonreacted sharp edges of the biomass bottom ash particles incorporated in the matrix of the binder were detected (Figure 2a). This statement is consistent with the EDX measurement (Figure 2e). In particle A, calcium, silicon, potassium, magnesium and iron dominate. These findings are consistent with the XRF analysis (Table 1). During the alkali activation, some of these particles were partially reacted (Figure 2b). New formed crystals were assigned to CSH/CASH and had foil-like shape, lamellar crystals [26]. When zeolitic waste was incorporated in the system, the particles of biomass bottom ash were almost not detected (Figure 2c). In this case, round-shaped particles could be detected and assigned to zeolitic waste [27]. The particles of zeolitic waste consisted of aluminum and silicon, with an inserted insignificant amount of sodium and potassium (Figure 2f). The incorporation of more zeolitic waste (15%) was connected with a slightly lower compressive strength (7.0 MPa). In this case, not all zeolitic waste participated in the alkali activation reactions. Some of it remained unreacted, as detected by SEM [28]. Large, round zeolitic waste crystals were surrounded by a lamellar CASH matrix, as shown in Figure 2d. In this matrix, the crystals that were specific to hydrosodalite were incorporated [29]. These crystals had thread ball-like spheres [30]. According to the EDX analysis, silicon, aluminum, calcium and sodium dominated (Figure 2g). 

## 3. Materials and Methods

**Figure 5:**
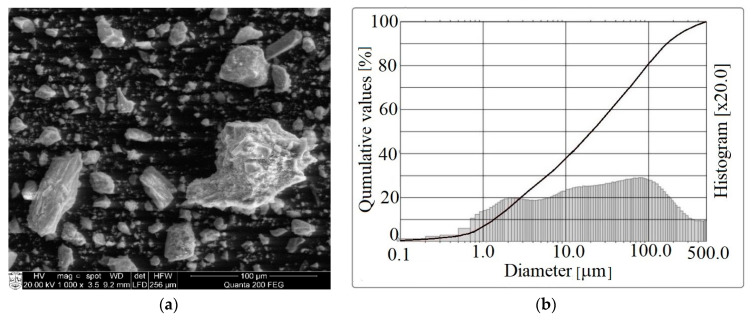
The SEM analysis (**a**) and granulometric composition (**b**) of biomass bottom ash.

Biomass bottom ash was used as the alkali-activated binder for the aluminosilicate precursor. In this research, biomass bottom ash (BBA) from Lithuanian (location Radviliškis) biofuel combustion plant was used. Its microstructure is sharp-edged (Figure 5a). The granulometric composition of the ash was also evaluated (Figure 5b). The ash particle size varied in a wide range of 1.0–500 µm. 

**Figure 6:**
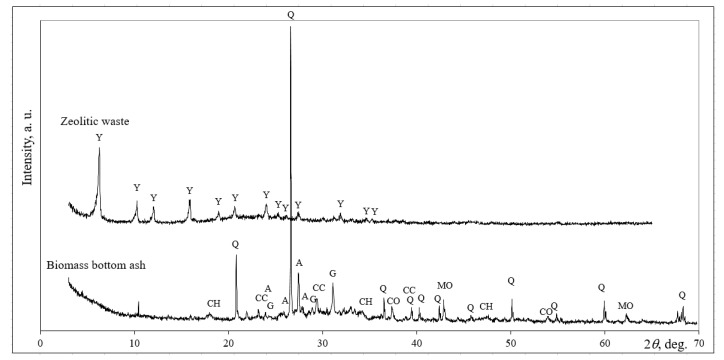
Mineral composition of initial aluminosilicate materials. Notes: Q—quartz SiO_2_ (77–1070), CH—calcium hydroxide Ca(OH)_2_ (84–1271), CC—calcium carbonate Ca(CO)_3_ (72–1652), A—anorthoclase (Na,K)(Si_3_Al)O_8_ (75–1631), G—gehlenite Ca_2_ Al(AlSiO_7_) (79–2421), CO—calcium oxide CaO (4–777), MO—magnesium oxide MgO (78–430) and Y—aluminum silicon hydrate Al_60.352_∙Si_139_∙O_371.52_∙H_5.984_ (73-2313).

The mineral composition of biomass bottom ash and zeolitic waste was evaluated using XRD analysis. Quartz, calcium hydroxide, calcium carbonate, anorthoclase, gehlenite, calcium oxide and magnesium oxide were found in the biomass bottom ash (Figure 6). The zeolitic waste has only one crystalline phase: zeoltite Y.

**Figure 7:**
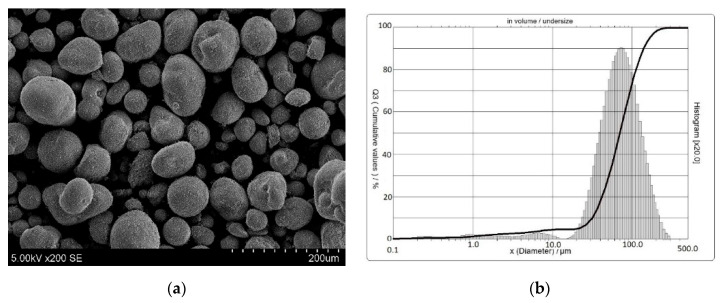
The SEM analysis (**a**) and granulometric composition (**b**) of zeolitic waste.

Zeolitic waste (FCC) was incorporated in the blends as the aluminosilicate material. In this research, the waste fluid catalytic cracking from oil refinery Orlen Lietuva in location Mažeikiai (Lithuania) was used. The SEM of zeolitic waste is shown in Figure 7a. Its particles are shaped similar to a ball. The particle size range is 19.80–177.81 μm (Figure 7b).

As shown in the oxide composition (Table 1), in the biomass bottom ash, the CaO (44.0%) percentage was the largest, followed by SiO_2_ (22.4%), MgO (8.29%) and K_2_O (8.69%). In the zeolitic waste, Al_2_O_3_ and SiO_2_ dominated with small amount of impurities. Tashima et al. [27] investigated this type of zeolitic residue as an alternative aluminosilicate material for alkali activation. In this work, the zeolitic waste increased the amounts of Al_2_O_3_ and SiO_2_ in the initial materials blends. Thus, this oxide composition became suitable for the precursor of an alkali-activated binder, as there dominated CaO, Al_2_O_3_ and SiO_2_. Before this zeolitic waste was incorporated in the alkali-activated matrix, it was used for ammonium sorption in the water environment; thus, 3.87% NH_4_Cl was detected in this zeolitic waste. The amount of NH_4_Cl was calculated according the amount of chloride in the FCC (Table 1).

Sodium hydroxide was used as the alkali activator. Commercial sodium hydroxide NaOH pellets (99% purity—Sigma-Aldrich, Steinheim, Germany) were used to prepare the alkaline solutions.

Six types of alkali-activated biomass bottom ash blends with varying amount of zeolitic waste were prepared (Table 2). The SiO_2_/Na_2_O molar ratio was stable in all mixtures. The amount of biomass bottom ash was substituted with zeolitic waste from 1 to 15 wt%. First, all dry aluminosilicate components were mixed. Then, the alkaline solution was filed, and pastes were mixed again to a homogenous mass for 4–5 min. 

The relatively slump results of the cement paste are shown in Table 2. It could be concluded that the powder of the ammonium-bearing zeolitic waste increased the relatively slump. The relative slump of the reference sample was 1.2, while the incorporation of FCC in this system led to an increase it until 1.98. The increase in the relative slump was 65% when the FCC content was 15% in the paste. Although the substitution of Portland cement by zeolitic waste reduced the slump [31], in this study, the values of it increased. Krerkchaiwan et al. [32] gave a similar tendency of flow by using ammonium salts in cement systems. 

The pastes of alkali-activated biomass bottom ash were poured into (20 × 20 × 20 mm) plastic molds. These samples were hydrated for 24 h in ambient laboratory conditions at 60 °C for another 24 h and again in ambient laboratory conditions for 26 days. The samples were placed into sealed bags to prevent drying.

A DRON-6 X-ray diffractometer was used to determine the mineral composition. It has Bragg-Brentano geometry using Ni-filtered Cu Kα radiation and a graphite monochromator, which operates at the voltage of 30 kV and an emission current of 20 mA. The step-scan covered an angular range of 2–70° in steps of 2 = 0.02°. 

Fourier-transform infrared (FTIR) spectrometry was conducted with a Perkin Elmer FTIR System spectrometer. One milligram of the substance was mixed with 200 mg of KBr and compressed in a forming press under vacuum for the IR analysis.

The XRFA analysis of the aluminosilicate precursor was performed using a fluorescence spectrometer S8 Tiger (Bruker AXS, Karlsruhe, Germany) operating at the counter gas helium 2 bar. 

The microscopic analyses such as scanning electron microscopy (SEM) of zeolitic waste, biomass bottom ash and the alkali-activated binder were performed by the high-resolution scanning electron microscope Hitachi S-3400N. The chemical compositions and their relative proportions (atomic % for example) of the samples were studied using energy-dispersive X-ray spectroscopy (EDX; BRUKER Quantax).

The flowability of the investigated pastes was evaluated by using a mini-slump test. In this investigation method was used the truncated conical mold, which size was 70 mm and 100 mm in diameter, and its height was 50 mm. The values of the relative slump were calculated according to Equation (1):

(1) $$\mathrm{RS}={\left(\frac{d}{d_{0}}\right)}^{2}-1$$

where RS is the relative slump, *d* is the average of two measured diameters of the paste spread and *d*_0_ is the bottom diameter of the conical cone, equal to 100 mm in this study [33].

The particle size distribution of zeolitic waste and biomass bottom ash was determined by the laser particle size analyzer Cilas 1090 LD.

After 7 and 28 days of hydration, the compressive strength of the samples was tested by using the hydraulic press ToniTechnik 2020. At least three samples were tested of each type. The compressive strength of the samples was determined in accordance to BS EN 196-1:2005.

## 4. Conclusions

The use of additional amounts of silica and alumina from zeolitic waste showed satisfactory results in the formation of alkali activation products (calcium silicate hydrate, calcium aluminum silicate hydrate and calcium sodium aluminum silicate hydrate) during hydration. These new hydration products are responsible for the strength development of the samples. The highest compression strength (14.7 MPa) was reached for samples with 3% of zeolitic waste, while the samples without zeolitic waste had 8.5 MPa. The compressive strength of the low-reactive biomass bottom ash can be improved by incorporating zeolitic waste during the alkali activation. In the microstructure of alkali-activated biomass bottom ash with zeolitic waste, newly formed crystals can be detected, which are assigned to CSH/CASH and hydrosodalite. The ammonium compound (NH_4_Cl), which was incorporated in the zeolitic waste, did not negatively affect the compressive strength of alkali-activated BBA samples and increased the values of the relative slump. This increase was 65% when 15% of FCC was incorporated in the paste. Zeolitic waste improved the compressive strength of the samples, changed the mineral composition and modified the microstructure of the hydrated binders. This study provided an alternative aluminosilicate binary system: biomass bottom ash and zeolitic waste, which is suitable for alkali-activated binders. It was recommended to use up to 5% in the alkali-activated BBA systems.

## Figures and Tables

**Table 1:** Oxide composition of initial aluminosilicate materials: zeolitic waste (FCC) and biomass bottom ash (BBA) (according to XRF), wt. %.

	CaO	SiO_2_	MgO	K_2_O	Al_2_O_3_	Fe_2_O3	P_2_O_5_	La_2_O_3_	SO_3_	Cl	TiO2	Other	L. I. *
BBA	44.0	22.4	8.29	8.69	2.51	2.18	5.05	0.89	0.58	-	0.33	1.02	4.06
FCC	0.37	35.4	0.44	0.04	48.77	1.02	0.08	1.63	0.07	2.57	3.57	6.04	

L. I. *—the loss on ignition at 1000 °C.

**Table 2:** Alkali-activated biomass bottom ash blends’ mixing proportions.

No.	Zeolitic Waste, g	Biomass Bottom Ash, g	Sodium Hydroxide, g	Water, mL	SiO_2_/Na_2_O	Relative Slump
1.	0	100	20.91	22.87	2	1.2
2.	1	99	20.94	22.07	2	1.23
3.	3	97	20.01	27.4	2	1.26
4.	5	95	21.52	24.58	2	1.29
5.	10	90	21.70	28.00	2	1.52
6.	15	85	21.91	32.8	2	1.98
